# Virulence of three European highly pathogenic H7N1 and H7N7 avian influenza viruses in Pekin and Muscovy ducks

**DOI:** 10.1186/s12917-019-1899-4

**Published:** 2019-05-10

**Authors:** David Scheibner, Claudia Blaurock, Thomas C. Mettenleiter, Elsayed M. Abdelwhab

**Affiliations:** Institute of Molecular Virology and Cell Biology, Friedrich-Loeffler-Institut, Federal Research Institute for Animal Health, Südufer 10, 17493 Greifswald, Insel Riems Germany

**Keywords:** Ducks, Muscovy duck, Pekin duck, Highly pathogenic avian influenza virus, HPAIV, H7N7, H7N1, Virulence

## Abstract

**Background:**

There is paucity of data on the virulence of highly pathogenic (HP) avian influenza viruses (AIV) H7 in ducks compared to HPAIV H5. Here, the virulence of HPAIV H7N1 (designated H7N1-FPV34 and H7N1-It99) and H7N7 (designated H7N7-FPV27) was assessed in Pekin and/or Muscovy ducklings after intrachoanal (IC) or intramuscular (IM) infection.

**Results:**

The morbidity rate ranged from 60 to 100% and mortality rate from 20 to 80% depending on the duck species, virus strain and/or challenge route. All Muscovy ducklings inoculated IC with H7N7-FPV27 or H7N1-FPV34 exhibited mild to severe clinical signs resulting in the death of 2/10 and 8/10 ducklings, respectively. Also, 2/10 and 6/9 of inoculated Muscovy ducklings died after IC or IM infection with H7N1-It99, respectively. Moreover, 5/10 Pekin ducklings inoculated IC or IM with H7N1-It99 died. The level of virus detected in the oropharyngeal swabs was higher than in the cloacal swabs.

**Conclusion:**

Taken together, HPAIV H7 cause mortality and morbidity in Muscovy and Pekin ducklings. The severity of disease in Muscovy ducklings depended on the virus strain and/or route of infection. Preferential replication of the virus in the respiratory tract compared to the gut merits further investigation.

## Background

Avian influenza viruses (AIV) belong to the family *Orthomyxoviridae*. The genome of AIV contains eight gene segments, which encode at least 11 viral proteins. They are classified according to the antigenic properties of the hemagglutinin (HA) and neuraminidase (NA) proteins into 16 HA (H1 to H16) and 9 NA (N1 to N9) subtypes [1]. Wild aquatic birds are the reservoir for all AIV and they transmit the virus to domestic birds. All AIV subtypes are low pathogenic (LP) causing mild local infection with or without overt clinical signs. Some H5 and H7 viruses can exhibit a highly pathogenic (HP) phenotype, mostly in domestic birds, causing multiorgan dysfunction due to systemic replication of the virus [2]. Wild and domestic ducks play an important role as a reservoir for AIV since the infection is usually asymptomatic. However, unlike the high mortality generally caused by HPAIV in chickens, some studies showed that the susceptibility of ducks to HPAIV differs by duck species (e.g. Pekin, Mallards or Muscovy), infection route, and/or age of ducks (i.e. ducklings are more susceptible than adult ducks) [3–9]. Muscovy ducks are more vulnerable than Pekin ducks to HPAIV H5N1 due to differences in immune responses [3, 4, 8]. However, these studies had been conducted using H5 HPAIV. In contrast, only limited data are available on the virulence of European H7 viruses, particularly in Muscovy ducks.

HPAIV H7 were first isolated in Europe in the early 1900s and have frequently been detected in poultry and wild birds in several European countries in the last two decades. Historical outbreaks caused by several H7Nx viruses in Europe in 1902, 1927, 1934, 1980s as well as recent outbreaks in 1999, 2003 and 2015 were described [10–15]. There is little information, if any, on the virulence of these H7 viruses in ducks. Neurological disorders and high mortality were observed in Muscovy ducks during the HPAIV H7N1 outbreaks in Italy in 1999–2000 [16]. However, experimental data from Muscovy ducks are still lacking. Three-week-old Pekin ducks did not show clinical signs, weight loss and/or mortality after the challenge with two different Italian HPAIV H7N1 [17, 18].

The objective of this study was to compare the virulence of two historic HPAIV A/FPV/Dutch/27 (H7N7) (designated hereafter as H7N7-FPV27) and A/Germany/FPV/1934 (H7N1) (designated hereafter H7N1-FPV34) and the recent HPAIV A/chicken/Italy/445/1999 (H7N1) (designated hereafter H7N1-It99) in Muscovy ducks (*Cairina moschata*) after intrachoanal (IC) inoculation. Furthermore, the impact of route of infection and species of ducks on virulence of H7N1-It99 was studied in Muscovy ducks and Pekin ducks (*Anas platyrhynchos domesticus*) after IC or intramuscular (IM) infection. The IC route was recommended for studying the pathogenesis of AIV to simulate natural upper respiratory exposure/transmission and to ensure that each bird receives the full dose [19, 20]. Likewise, IM injection was recently used to assess the pathogenicity of HPAIV in ducklings, resembling intravenous pathogenicity index (IVPI) in chickens [9]. Previous studies have shown that all three viruses were highly lethal in chickens under experimental conditions [10, 21, 22].

## Results

In this study, the virulence of the two historic H7N7-FPV27 (group 1) and H7N1-FPV34 (group 2) in Muscovy ducks was assessed. The impact of duck species and inoculation route on virulence of H7N1-It99 was tested in Muscovy (groups 3 and 4) and Pekin ducks (groups 5 and 6) after IC or IM infection. All birds were observed for 10 days and clinically scored as 0 (healthy), 1 (sick), 2 (severely sick) or 3 (dead) and the pathogenicity index (PI) was calculated as a scale from 0 (avirulent) to 3 (highly virulent) [23].

### Clinical examination

**Fig. 1:**
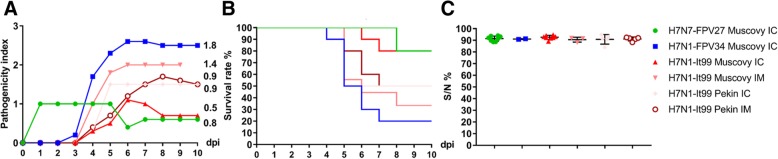
Pathogenicity index, survival rate and seroconversion in Muscovy and Pekin ducks after challenge with H7 viruses. Shown are the average clinical scores for each group. All birds were observed for 10 days and clinically scored according to the OIE recommendations as 0 (healthy), 1 (sick), 2 (severely sick) or 3 (dead) and the pathogenicity index (PI) was calculated as a scale from 0 (avirulent) to 3 (highly virulent). The PI, shown as numbers, was calculated by dividing the sum of the arithmetic mean values of daily scores by 10 (the number of observation days). Dead birds were score 3 until the end of the experiment according to the OIE recommendations (**a**). Survival rate in each group showing the daily mortality of ducks after infection (**b**) and antibody titers at the end of experiment using ELISA (expressed as mean ± standard deviation) (**c**)

After challenge, Muscovy ducks showed diarrhea and nervous signs including circling, rolling, incoordination, steady gait and/or opisthotonus starting from 2 dpi. The morbidity rate ranged from 60 to 100% and mortality from 20 to 80% (Fig. 1a and b).

All Muscovy ducklings inoculated with H7N7-FPV27 (group 1) showed mild depression at 1 dpi, which became more prominent at 2 dpi. All ducklings in this group returned to normal starting from 3 dpi except two ducklings that died or were killed for humane reasons by day 7 (Table 1) after showing severe depression and central nervous signs. Both birds were scored dead at 8 dpi. The PI value was 0.8 (Table 1).

**Table 1:** Clinical examination of Muscovy and Pekin ducks after challenge with H7 viruses

Group	Species	Challenge route	Virus	Morbidity (%)	Mortality (%)	MDT (range)	PI
1	Muscovy	IC	H7N7-FPV27	10/10 (100%)	2/10 (20%)	7 (7)	0.8
2	Muscovy	IC	H7N1-FPV34	10/10 (100%)	8/10 (80%)	5.4 (4–7)	1.8
3	Muscovy	IC	H7N1-It99	7/10 (70%)	2/10 (20%)	6.5 (6–7)	0.5
4	Muscovy	IM	H7N1- It99	9/9 (100%)	6/9 (66.7%)	5.7 (5–8)	1.4
5	Pekin	IC	H7N1- It99	6/10 (60%)	5/10 (50%)	5 (5)	0.9
6	Pekin	IM	H7N1- It99	8/10 (80%)	5/10 (50%)	6 (5–7)	0.9

Range refers to the day of first and last mortality

*IC* intrachoanal, *IM* intramuscular, *MDT* mean death time per day calculated for dead birds, *PI* Pathogenicity index

Muscovy ducklings challenged with H7N7-FPV34 (group 2) exhibited significantly (*p* < 0.01) more severe and prominent signs than ducklings in group 1 and group 3 with a PI of 1.8 (Table 1). At 3 dpi, two ducklings showed moderate nervous signs. At 4 dpi, one bird died and four birds were humanely killed due to severe nervous signs and scored as dead at 5 dpi. Likewise, at 6 dpi, two ducklings died and another duckling was killed and scored dead at 7 dpi. In total, eight out of ten ducklings died between 4 and 7 dpi with MDT of 5.4 days. Two birds survived, however showing mild to moderate clinical signs at 10 dpi (Fig. 1a and b).

Seven out of ten Muscovy ducklings inoculated IC with H7N1-It99 (group 3) showed neurological signs, while three ducklings remained healthy to the end of the experiment (PI of 0.5). Clinical signs started at 4 dpi. One bird showed mild to severe clinical signs from 4 dpi to 10 dpi. Meanwhile, four birds showed mild transient depression and recovered by 8 dpi. Two ducklings were killed at 5 and 6 dpi (and scored dead at the next day) for humane reasons after showing severe clinical signs (MDT = 6.5) (Table 1, Fig. 1a and b).

Muscovy ducklings inoculated IM with H7N1-It99 (group 4) showed higher morbidity and mortality compared to IC-inoculated animals (group 3). In total, six out of nine Muscovy ducklings inoculated IM with H7N1-It99 died with a PI of 1.4 and MDT of 5.7 days: three ducklings were killed at 4 dpi and one at 7 dpi. Two birds were found dead at 5 and 6 dpi. Two ducklings remained sick until 10 dpi and one bird recovered at 9 dpi (Fig. 1a and b).

Six out of ten Pekin ducklings inoculated IC with H7N1-It99 (group 5) showed clinical signs (PI 0.9) where five Pekin ducklings died at 5 dpi (Fig. 1a and b) with or without showing moderate depression and nervous signs. One bird had temporary torticollis; however, it accessed food and water easily until 10 dpi. The other four ducklings did not show any clinical signs.

Eight out of ten Pekin ducklings injected IM with H7N1-It99 (group 6) showed clinical signs (PI = 0.9) where five Pekin ducklings died at 5 dpi (*n* = 2), day 6 dpi (*n* = 2) and 7 dpi (*n* = 1) after showing mild to moderate nervous signs (Fig. 1a and b). Three ducklings showed transient mild to moderate depression two of which recovered after 2 days. Two ducklings did not show any clinical signs during the observation period. No statistical difference in clinical scoring of IM or IC inoculated ducks was observed (*P* > 0.99).

### Virus shedding

**Fig. 2:**
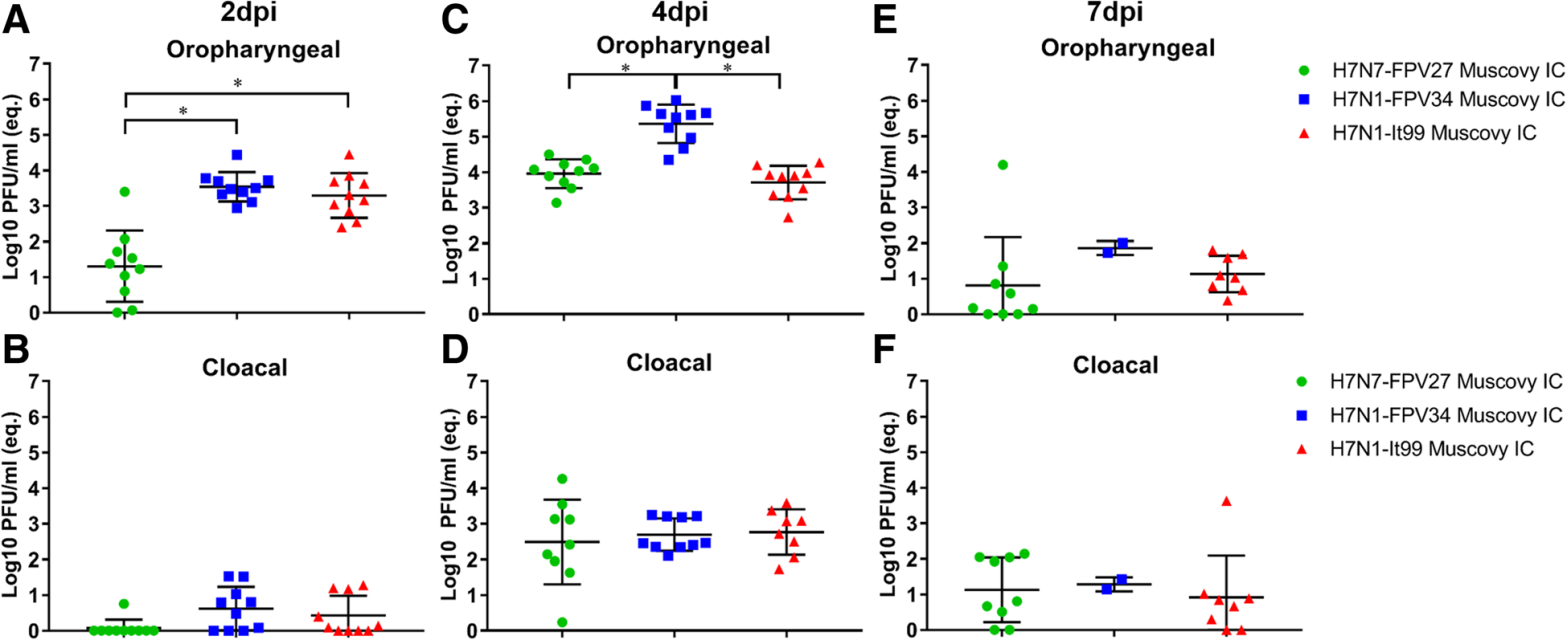
Virus excretion in oropharyngeal and cloacal swabs after intrachoanal inoculation of Muscovy ducks with HPAIV H7 viruses. Virus excretion in oropharyngeal and cloacal swabs at 2 (**a**-**b**), 4 (**c**-**d**) and 7 (**e**-**f**) days post inoculation using RT-qPCR expressed by equivalent Log10 PFU/mL. All results are expressed as average and standard deviation (mean ± SD) per group at indicated time points post infection. The viruses were not detected in swabs collected at 10 dpi (data not shown). Statistical significance shown in asterisks indicate *P* values < 0.05

**Fig. 3:**
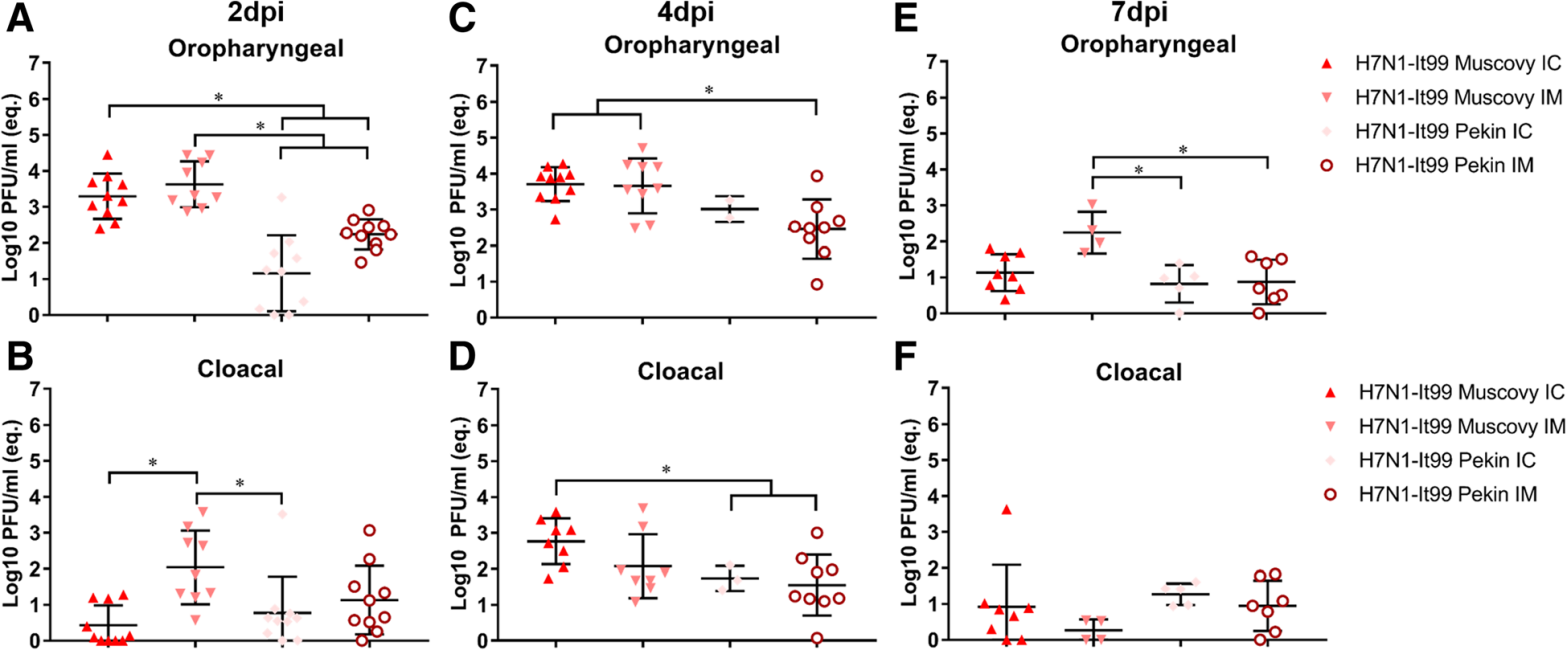
Virus excretion in the oropharyngeal and cloacal swabs in Muscovy and Pekin ducks challenged with H7N1-It99 by intrachoanal or intramuscular routes. Virus excretion in oropharyngeal and cloacal swabs at 2 (**a**-**b**), 4 (**c**-**d**) and 7 (**e**-**f**) days post inoculation using RT-qPCR expressed by equivalent Log10 PFU/mL. All results are expressed as average and standard deviation (mean ± SD) per group at indicated time points post infection. The viruses were not detected in swabs collected at 10 dpi (data not shown). Statistical significance shown in asterisks indicate *P* values < 0.05. For clarity, results of H7N1-It99 IC-inoculated ducks (group 3) was presented in Figure 3 again for comparison

Influenza virus RNA was not detected in swab samples collected before infection. Cloacal (CL) and oropharyngeal (OP) swabs collected from inoculated birds at 2, 4, 7 and 10 dpi were tested by real time RT-PCR. Results are summarized in Figs. 2 and 3. Swab samples collected at 10 dpi were negative (data not shown).

### IC-inoculated Muscovy ducks (groups 1, 2 and 3)

Virus excretion in the OP swabs was higher than in CL swabs particularly at 2 and 4 dpi (Fig. 2). The mean quantity of virus excreted in the OP and CL swabs at 4 dpi was higher than the quantity of virus excretion at 2 and 7 dpi (Fig. 2). At 2 dpi, the virus was detected in OP swabs in all ducks, except 2 Muscovy ducks inoculated with H7N7-FPV27 (group 1) (Fig. 2a). The latter virus was excreted in OP swabs at significantly lower amounts than H7N1-FPV34 (group 2) and H7N1-It99 (group 3) (*P* < 0.001) at 2 dpi (Fig. 2a). At 4 dpi, H7N1-FPV34 was shed with significantly higher amounts in the OP swabs than the other two viruses (Fig. 2c), while at 7 dpi (Fig. 2e) and in CL swabs at each time all viruses were excreted at comparably similar levels (Fig. 2b, d and f).

### Muscovy ducks (groups 3 and 4) and Pekin ducks (groups 5 and 6) challenged with H7N1-It99

The mean quantity of virus excretion in the OP swabs was higher than in CL swabs particularly at 2 and 4 dpi regardless of duck species or inoculation route (Fig. 3a-d). In the OP swabs, there was no significant difference in the amount of H7N1-It99 excreted from Muscovy (groups 3 and 4) or Pekin ducklings (groups 5 and 6) after IM or IC challenge at each time point. The mean quantity of virus excreted in the OP swabs by Muscovy ducks was higher than the virus excreted by Pekin ducks (Fig. 3).

### Seroconversion

All serum samples collected before infection were negative for anti-influenza antibodies. At the end of the experiment, all surviving birds possessed anti-NP antibodies detectable by ELISA. There was no significant difference in antibody titers between the different groups of ducklings regardless of the virus subtype, species or inoculation route (Fig. 1c).

## Discussion

Ducks play an important role as a reservoir for AIV including HPAIV [8, 24]. However, data on the susceptibility of Pekin ducks are scarce and no data on the susceptibility of Muscovy ducks to H7 viruses particularly from Europe are available. In Muscovy ducklings, the historic H7N1-FPV34 was more virulent than historic H7N7-FPV27 and contemporary H7N1-It99 after IC inoculation as indicated by higher morbidity and mortality. This is partially in agreement with findings in a previous historic study [10] which showed that 9/10 intranasally inoculated two-week-old Khaki Campbell ducklings, an egg-laying duck breed, with H7N1-FPV34 exhibited clinical signs and 2/10 ducklings died at 7 and 11 dpi while H7N7-FPV27 did not cause any clinical signs or mortality [10].

Muscovy ducklings infected with H7N7-It99 showed more severe symptoms than IC inoculated animals, while no impact of the infection route on virulence was observed in Pekin ducklings. In a previous study, intranasal, intrachoanal or ocular infection with an HPAIV H5N1 produced similar outcome in two-week-old Pekin or Muscovy ducks with an HPAIV H5N1 [8]. C Grund, et al. [9] reported higher mortality in Pekin and Muscovy ducks after IM injection than oculonasal inoculation with HPAIV H5N8. Mortality in Mallard ducks after intravenous injection was strain-dependent (i.e. some HPAIV H5N1 induced 100% mortality while others were avirulent) [25].

In the current study, five of ten Pekin ducklings died after IC or IM infection with H7N1-It99, whereas inoculation of three-week-old Pekin ducks with two different Italian HPAIV H7N1 induced no mortality [17, 18]. Here, we used ten-day-old ducklings because it has been shown that younger ducklings are more susceptible to some HPAIV H5N1 than adult ducks [26, 27]. Differences in the age of Pekin ducks as well as the use of different virus strains may explain the higher mortality in Pekin ducks in the current study compared to previous studies using HPAIV H5 [17, 18, 26].

Muscovy ducks are more vulnerable than Pekin ducks to some H5N1 viruses [6, 8, 9]. On the other hand, mortality was observed only in Pekin but not in Muscovy ducklings after challenge with an HPAIV H5N1 [7]. So far, no data are available on the susceptibility of Muscovy ducks to HPAIV H7. Recently, we have shown that a German HPAIV H7N7 did not result in any mortality in Pekin or in Muscovy ducks (Scheibner et al. submitted). In the current study, H7N1-It99 induced a lower mortality rate in Muscovy ducklings inoculated IC (20%) than Pekin ducklings inoculated by the same route (50%). Interestingly, Muscovy ducklings excreted H7N1-It99 virus at significantly higher levels than Pekin ducklings indicating important role in spreading of the virus into the environment. Therefore, it is important to consider subtype/strain variations in the assessment of virulence of HPAIV H7 in different duck species.

AIV preferentially replicate in the digestive tract of ducks, which may enable continuous shedding of the virus into the environment (i.e. water ponds) [28–30]. Interestingly, our results indicated that although all viruses were excreted in OP and CL swabs at 2, 4 and 7 dpi, the amount in OP swabs was higher than in CL swabs regardless of the challenge virus, duck species or route of infection. Similar results were observed after experimental infection of Mallard ducks with HPAIV Tk/Italy/99 (H7N1), Ck/Netherlands/03 (H7N7), Ck/North Korea/05 (H7N7) and Ck/Victoria/85 (H7N7). Conversely, HPAIV Ck/Jalisco/12 (H7N3) and Ck/Canada/05 (H7N3) were excreted in a higher amount in the CL than in OP swabs [24]. The preferential pattern of virus excretion of H7 viruses from the oropharynx merits further investigation.

## Conclusions

Taken together, the three European H7 viruses used in this study exhibited variable virulence in Muscovy ducklings. H7N1-FPV34 induced 80% mortality, while H7N7-FPV27 and H7N1-It99 killed only 20% of IC-inoculated ducklings. H7N1-It99 exhibited higher virulence in IM-injected Muscovy than in IC inoculated ducklings with 66.7% and 20 mortality, respectively. Furthermore, H7N1-It99 exhibited moderate virulence in Pekin ducklings with no difference between the IC or IM inoculation routes. Moreover, regardless of the challenge route, Muscovy ducks excreted higher amounts of H7N1-It99 than Pekin ducks. Findings in this study showed the variable virulence of HPAIV H7 in different duck species.

## Methods

The main goal of this study was to assess the virulence of three European H7 viruses in domestic ducks. Muscovy and Pekin ducklings were inoculated via the intrachoanal and/or intramuscular and were observed for 10 days post inoculation/injection (dpi). Swab samples were collected from all ducklings at 2, 4, 7 and 10 dpi and tested by generic real time RT-PCR.

### Virus propagation

Viruses in this study were kindly provided by Timm C. Harder. All viruses were propagated in the allantoic sac of specific pathogen free (SPF) embryonated chicken eggs (ECE) (VALO BioMedia GmbH) according to the standard protocol [23]. Allantoic fluid was collected and the hemagglutination activity was measured using 1% chicken erythrocytes [23]. Aliquots of virus stocks were kept at − 70 °C until use. All viruses were propagated and handled in biosafety level 3 laboratory at the FLI.

### Virus titration

Virus titration was done using plaque assay. Confluent MDCKII cells in 12-well plates were infected with ten-fold serial dilutions of specified viruses for an hour at 37 °C/5% CO_2_. Cells were overlaid with semisolid BactoTM Agar (BD) containing minimal essential medium (MEM) and 4% bovine serum albumin (BSA) (MP Biomedicals). All plates were incubated for 3 days at 37 °C. Cells were fixed by 10% formaldehyde containing 0.1% crystal violet. Plaques were counted and viral titers were expressed as plaque forming units per ml (PFU/ml).

### Animal experiment

Animal experiments were carried out after approval by the authorized ethics committee of the State Office of Agriculture, Food Safety, and Fishery in Mecklenburg – Western Pomerania (No. 7221.3-1-060/17) and approval by the commissioner for animal welfare at the FLI representing the Institutional Animal Care and Use Committee (IACUC) following the German Regulations for Animal Welfare.

One-day old commercial Pekin and Muscovy ducklings were purchased from Czarkowski GbR, Storkow, Germany. At the FLI, swab samples were collected from all ducks and examined to exclude infection by influenza [31] and Salmonella spp. [32, 33]. Birds were housed in different groups and food and water were added ad-libitum. At 10 days of age, male and female Pekin and Muscovy ducklings were randomly allocated to separate groups. At day 0, ten birds were inoculated with 0.2 mL containing 5 × 10^5^ PFU via the IC or IM route as described in Table 1. All animals were observed for 10 days and clinically scored [21]. Briefly, healthy ducks were given score (0), sick birds showing one clinical sign (e.g. depression, diarrhea, nervous manifestations, respiratory disorders) were given score (1), severely sick birds showed more than one clinical sign were given score (2) and dead birds were given score (3). The pathogenicity index (PI) was calculated by dividing the sum of the arithmetic mean values of daily scores by 10 (the number of observation days). The PI for each virus ranged from 0 (avirulent) to 3 (highly virulent) [23]. Serum and swab samples were collected and stored in BSL-3 laboratories, at − 20° and − 70 °C, respectively.

### Virus excretion

OP and CL swabs were collected before infection and at 2, 4, 7 and 10 days post inoculation/injection (dpi) on swab media and stored at − 70 °C until use. Swabs medium contained (pro liter) MEM Eagle (Sigma-Aldrich), 5.6 mL BSA (MP Biomedicals) and antibiotics (1% enrofloxacin, 0.5% lincomycin and 0.1% gentamycin). The RNA was extracted from swab media using NucleoMagVet® 8/96 PCR Clean-up Core Kit (Macherey & Nagel GmbH, Germany) in KingFisher Flex Purification System (Thermo Fisher Scientific, USA). The quantity of virus excretion in swab samples was determined using SuperScript™ III Platinum™ One-Step qRT-PCR Kit (Invitrogen, Germany) according to the manufacturer guidelines and generic real-time reverse-transcription polymerase chain reaction (RT-qPCR) [31]. The RT-qPCR reactions were performed in AriaMx Real-time PCR System (Agilent, Germany). For RT-PCR amplification the following thermal profile was applied: 30 min at 50 °C (reverse transcription) and 15 min at 94 °C (inactivation of the reverse transcriptase/activation Taq polymerase), followed by 42 cycles at 94 °C for 30s (denaturation), 58 °C for 30s (annealing) and 68 °C for 30s (elongation). Standard curves using HPAIV H7N7 (10^1^ to 10^6^ pfu/mL) were run in each RT-qPCR round. The relative amount of excreted virus was quantified by plotting the Ct-values in the standard curves and results are expressed as average ± standard deviation equivalent log_10_ PFU/ml.

### Serological examination

Blood was collected before infection (5 samples pro species) via wing vein puncture and at the end of the experiments from all surviving ducks after euthanization using isoflurane® (CP-Pharma). Briefly, the ducklings were gently and carefully put inside a tightly close beaker containing four to five isoflurane-soaked gauze sponges for about 2 min to ensure deep anesthesia. Complete loss of consciousness was achieved as assessed by complete suppression of pedal and ocular reflexes. Ducklings were taken out and whole blood was collected in 50 mL Falcon tubes via cutting the jugular vein using knife. Thereafter, the head was separated from the body rapidly and completely. The serum was separated from the blood after 24 h incubation in the fridge followed by centrifugation and inactivation at 72 °*C. sera* were tested for anti-AIV nucleoprotein (NP) using enzyme-linked immunosorbent assay (ELISA) by ID screen Influenza A Antibody Competition Multispecies kit (IDvet). Plates were read in Tecan® ELISA reader. The cut-off point according to the manufacture guideline was 55%, samples between 45 and 55% were considered questionable and samples lower than 45% were considered negative.

### Statistics

Statistical differences were analyzed using non-parametric Kruskal-Wallis and Mann-Whitney Wilcoxon tests with post hoc Tukey tests. Results were considered statistically significant by any test at *p* value < 0.05. All analysis was done by GraphPad Prism software. Clinical scoring for mean values for each bird in 10 day-observation period was compared.

## Abbreviations

AIV: Avian influenza viruses
BSA: Bovine serum albumin
CL: Cloacal
dpi: Days post inoculation/injection
ECE: Embryonated chicken eggs
ELISA: Enzyme linked immunosorbent assay
FLI: Friedrich-Loeffler-Institut
HA: Hemagglutinin
HP: Highly pathogenic
IACUC: Institutional Animal Care and Use Committee
IC: Intrachoanal
IM: Intramuscular
IVPI: Intravenous pathogenicity index
LP: Low pathogenic
MDCKII: Madin-Darby canine kidney cells type II
MDT: Mean death time
MEM: Minimum essential medium
NA: Neuraminidase
NP: Nucleoprotein
OP: Oropharyngeal
PCR: Polymerase chain reaction
PFU/mL: Plaque forming units per ml
RT-qPCR: Real-time reverse-transcription polymerase chain reaction
SPF: Specific pathogen free
